# Case Report: Immune checkpoint inhibitor-induced gastrointestinal toxicity: the diagnostic challenge of sequential ileus and colitis

**DOI:** 10.3389/fmed.2026.1966665

**Published:** 2026-09-24

**Authors:** Mouhamad Ismail, Nicola Thompson, Brent O’Carrigan, Philippa Corrie

**Affiliations:** Department of Oncology, Cambridge University Hospital, Cambridge, United Kingdom

**Keywords:** colitis, ileus, immunotherapy, immunotherapy toxicity, melanoma

## Abstract

**Introduction:**

Immune checkpoint inhibitors can cause diverse immune-related adverse events, the early recognition of which is critical. Intestinal pseudo-obstruction caused by immune-mediated gastrointestinal dysautonomia is a rare, potentially fatal, and under-recognized toxicity that may be difficult to distinguish from more common gastrointestinal complications.

**Methods:**

We describe the clinical course, investigation, and management of a patient with metastatic esophageal mucosal melanoma who developed sequential gastrointestinal toxicities following combination ipilimumab and nivolumab therapy, focusing on early recognition and the diagnostic challenges associated with recognizing immune-mediated intestinal dysmotility.

**Results:**

Following four cycles of immunotherapy, the patient developed intractable nausea, vomiting, and constipation. Computed tomography demonstrated diffuse small bowel dilatation without a mechanical transition point, raising suspicion of immune checkpoint inhibitor-induced ileus. Despite intravenous corticosteroids, the clinical phenotype evolved over the following week to high-frequency diarrhea, with repeat imaging and endoscopy supporting superimposed enterocolitis. Following an initial response to prolonged corticosteroid treatment, diarrhea recurred and ultimately improved after infliximab therapy. The sequential presentation of dysmotility and inflammatory colitis complicated the assessment of disease evolution and decisions regarding the escalation of immunosuppression.

**Discussion:**

This case highlights intestinal pseudo-obstruction as an important but easily overlooked immune-related adverse event and demonstrates how rare gastrointestinal dysautonomia may precede, coexist with, or be masked by more familiar immune-mediated colitis. Recognition of the contrasting clinical phenotypes, particularly otherwise unexplained nausea, vomiting, constipation, and bowel dilatation without mechanical obstruction, is essential because the underlying mechanisms and therapeutic responses may differ. Greater awareness of these early warning features, coupled with prompt investigation and multidisciplinary assessment, may reduce diagnostic delays and enable earlier treatment of this potentially life-threatening toxicity**.**

## 1 Introduction

Mucosal melanoma is a malignancy with high morbidity and mortality (1). Despite recent improvements in the management of cutaneous melanoma with the introduction of immune checkpoint inhibitors (ICIs), patients diagnosed with mucosal melanoma experience inferior outcomes with ICIs while suffering similar rates of immune-related adverse events (irAEs) (1). Colitis is one of the most common irAEs, but intestinal pseudo-obstruction (ileus) is a rare irAE that is usually refractory to corticosteroids (2). In this case report, we present a case of sequential ileus followed by colitis secondary to combination ICI therapy (ipilimumab and nivolumab), aiming to raise awareness of this irAE and highlighting the challenge of early detection with overlapping toxicities and the diagnostic dilemmas associated with the management of rare irAEs.

## 2 Case presentation

A 57-year-old male patient presented in November 2025 with a 1-month history of progressive dysphagia, weight loss, and retrosternal chest pain. His medical history included clinical depression, and he had complex social circumstances, living in sheltered accommodation for the previous 3 months. Computed tomography (CT) scans of the chest, abdomen, and pelvis revealed a 6.5-cm mural thickening of the mid-esophagus. An endoscopic biopsy confirmed a poorly differentiated high-grade malignancy, with immunohistochemical markers consistent with mucosal melanoma. Staging via positron emission tomography demonstrated intense fluorodeoxyglucose avidity in the esophageal mass, with metastatic involvement of the left upper paratracheal, para-esophageal, and right cervical (level IV) nodes. A metabolically active nodule in the right chest wall was also identified. The patient was started on combination immunotherapy with ipilimumab (CTLA-4 inhibitor) and nivolumab (PD-1 inhibitor) in January 2026.

Following the second cycle, the patient presented with grade 2 fatigue and a syncopal episode. Biochemical evaluation confirmed ICI-induced secondary hypoadrenalism. He was managed with an initial dose of 100 mg IV of hydrocortisone, followed by a maintenance regimen (20/10/10 mg for 2 weeks, transitioned to a lifelong replacement dose of 10/5/5 mg). He suffered two subsequent admissions for adrenal crisis secondary to medication non-compliance and linked to social instability, which resolved rapidly with intravenous corticosteroid resuscitation and further endocrine counseling.

Following cycle 4, the patient presented with intractable nausea, vomiting, and constipation. Blood tests, including electrolyte, metabolic, and endocrine panels, were normal apart from a stage 1 acute kidney injury. Infectious causes of his gastrointestinal symptoms were excluded through appropriate microbiological investigations. A neurological examination was not undertaken at the time. A CT scan showed diffuse small bowel dilatation without a mechanical transition zone, suggesting ileus. With no other cause identified, the findings were attributed to ICI toxicity. Treatment was initiated with 1 mg/kg IV methylprednisolone. On day 4, his bowel function started to improve, and by day 7 of admission, the clinical picture had evolved into high-frequency grade 3 diarrhea. A repeat CT showed a dilated right hemi-colon (caecum 7 cm) with diffuse mesenteric fat stranding. Flexible sigmoidoscopy noted erythema and mild inflammation. Biopsies revealed mild chronic proctocolitis. The patient was now managed as having ICI-induced enterocolitis. After 14 days of IV methylprednisolone, his diarrhea had improved sufficiently to allow discharge on a tapered course of oral corticosteroids.

Forty-eight hours post-discharge, the patient returned to the Emergency Department with profound weakness and hypotension. Laboratory results were diagnostic of diabetic ketoacidosis (DKA). He was stabilized using a standard DKA insulin protocol. Investigations, including an autoimmune antibody panel, suggested that steroid-induced hyperglycemia was more likely than ICI-related type 1 diabetes mellitus. During the same admission, the patient experienced a recurrence of diarrhea. He received infliximab (5 mg/kg) therapy for steroid-refractory colitis while continuing a slow corticosteroid wean, and his bowel function improved significantly, returning to baseline.

## 3 Discussion

This case highlights the challenges of early recognition of immunotherapy toxicities, particularly when ICI-induced ileus precedes the development of overt enterocolitis. Gastrointestinal (GI) toxicities are among the most common irAEs, with diarrhea occurring in up to 54% of patients receiving anti-CTLA-4 therapy and severe enterocolitis requiring treatment in approximately 10% of patients on CTLA-4 inhibitors (2). However, ICI-induced intestinal pseudo-obstruction secondary to gastrointestinal dysautonomia is a rare and under-recognized entity. The National Comprehensive Cancer Network (NCCN) guidelines acknowledge that GI tract paresis due to myenteric neuritis is a rare toxicity associated with ICI therapy (3).

The pathophysiology of ICI-induced ileus is fundamentally different from that of ICI-induced colitis. While colitis results from immune-mediated mucosal inflammation with CD8+ T-cell infiltration and disruption of intestinal homeostasis, ICI-induced ileus arises from immune-mediated injury to the enteric nervous system, specifically the myenteric (Auerbach's) and submucosal (Meissner's) plexuses (4, 5). In one case of ICI-induced ileus where an autopsy was undertaken, no staining of myenteric ganglion cell bodies or intramuscular nerves was seen, supporting extensive loss of ganglion cells within the myenteric plexus (6).

A recent systematic review by Sennfält et al. identified only 18 individual cases of ICI-induced GI dysautonomia in the literature, with a mean onset of 13.6 weeks from ICI exposure (5). Critically, this entity carries a high mortality rate of 47%, and corticosteroids were used only in 72% of cases, often at subtherapeutic doses and for insufficient durations (5). The one patient in their case series who achieved full recovery received prolonged high-dose corticosteroids, followed by mycophenolate mofetil (MMF) maintenance therapy, suggesting that extended immunosuppression may be necessary. That study emphasized that early recognition and effective immunosuppression are essential to optimize outcomes (5). In our case, the initial presentation of nausea, vomiting, and constipation without a mechanical transition point on CT was consistent with ICI-induced ileus, and the subsequent evolution to high-frequency diarrhea with radiological and endoscopic evidence of colitis suggested that these may either represent a continuum of immune-mediated gastrointestinal injury or that the initial ileus may have masked the development of colitis, potentially delaying appropriate escalation of immunosuppressive therapy.

Current management guidelines for ICI-induced ileus are extrapolated from the broader literature on neurologic irAEs and autonomic neuropathy, as prospective data specific to this entity are lacking. The NCCN guidelines recommend the early addition of high-dose steroids and a multidisciplinary team approach when gastrointestinal tract paresis due to myenteric neuritis is suspected (3). However, the optimal dose, route, and duration of corticosteroid therapy remain undefined (3).

Unlike ICI colitis, where infliximab and vedolizumab have demonstrated efficacy in steroid-refractory cases, the role of biologic agents in ICI-induced ileus is unclear. The gut-selective mechanism of vedolizumab (anti-integrin α4β7) targets mucosal inflammation but would not be expected to address neuronal injury (7). Similarly, infliximab's efficacy in colitis is predicated on blocking TNF-α-mediated mucosal inflammation (7). In the case of ICI-induced ileus, there is a lack of evidence for second-line treatments following corticosteroids, but further immunosuppression with MMF, intravenous immunoglobulin, or tacrolimus has been reported with limited success (3–5).

## 4 Conclusion

ICI-induced intestinal pseudo-obstruction secondary to myenteric plexopathy is a rare, under-recognized, and potentially fatal gastrointestinal irAE that is pathophysiologically and clinically distinct from ICI-induced colitis. This case illustrates the diagnostic challenges posed by the sequential ileus and colitis and highlights the critical importance of early recognition and aggressive immunosuppression. Clinicians should maintain a low threshold for initiating high-dose corticosteroids when ICI-induced ileus is suspected and should anticipate the need for prolonged immunosuppression and multidisciplinary care. Further research is urgently needed to define optimal treatment strategies and to identify biomarkers that can predict this potentially life-threatening complication.

## Data Availability

The original contributions presented in this study are included in the article/Supplementary Material; further inquiries can be directed to the corresponding author.
